# Gingival inflammation and leukocyte–endothelium cell interactions in women with polycystic ovary syndrome

**DOI:** 10.1002/JPER.24-0148

**Published:** 2024-10-15

**Authors:** Cecilia Fabiana Márquez‐Arrico, Francisco Javier Silvestre, Meylin Fernández‐Reyes, Sandra López‐Domènech, Jonathan Hermenejildo, Zaida Abad‐Jiménez, Javier Silvestre‐Rangil, Pablo Fernández‐Collazo, Carlos Morillas, José María Montiel‐Company, Víctor M. Víctor, Milagros Rocha

**Affiliations:** ^1^ Department of Stomatology University of Valencia Valencia Spain; ^2^ Department of Stomatology University Hospital Doctor Peset Foundation for the Promotion of Health and Biomedical Research Valencia Spain; ^3^ Department of Endocrinology and Nutrition University Hospital Doctor Peset Foundation for the Promotion of Health and Biomedical Research Valencia Spain; ^4^ Department of Pharmacology University of Valencia Valencia Spain; ^5^ Department of Physiology University of Valencia Biomedical Research Institute Valencia (INCLIVA) Valencia Spain; ^6^ National Network of Biomedical Research on Hepatic and Digestive Diseases CIBERehd Valencia Spain

**Keywords:** atherosclerosis, endothelial dysfunction, inflammation, neutrophils, periodontal diseases, polycystic ovary syndrome

## Abstract

**Background:**

Given the link between chronic inflammation and periodontal pathologies and increased cardiovascular risk, this study aims to investigate if gingivitis exacerbates the inflammatory response and subclinical atherosclerotic markers in women with polycystic ovary syndrome (PCOS).

**Methods:**

For this case–control study, women were assigned to three groups: two PCOS groups (with and without gingivitis) and a control group. Anthropometric and biochemical variables were determined, along with periodontal parameters (probing pocket depth [PPD], clinical attachment level [CAL], bleeding on probing [BOP], plaque index, calculus index, and tooth loss), systemic and neutrophil inflammatory markers (tumor necrosis factor alpha [TNFα], C‐reactive protein [CRP], and c‐Jun N‐terminal kinase [JNK]), systemic oxidative stress mediators (myeloperoxidase [MPO] and glutathione), soluble cellular adhesion molecules, and neutrophil–endothelium cell interactions (rolling flux, velocity, and adhesion).

**Results:**

Of 104 women recruited, 68 had PCOS, 24 of whom presented gingivitis, and 36 were controls. PCOS patients presented altered sexual hormone, lipid, and carbohydrate profiles. Levels of systemic inflammatory markers, MPO, and soluble platelet selectin (sP‐selectin) were higher, and glutathione levels were lower in PCOS patients. BOP, plaque, and calculus index values were higher in PCOS patients with gingivitis. Neutrophils from PCOS patients showed increased JNK and decreased adhesion under flow conditions, with reduced rolling velocity and increased rolling flux and cellular adhesion, all of which were more pronounced in those with gingivitis. BOP was independently associated with rolling velocity, rolling flux, and cellular adhesion.

**Conclusion:**

Neutrophils of PCOS patients with gingivitis exhibit hyperactivity, promoting interaction with the endothelium and potentially contributing to atherosclerotic disease.

**Plain Language Summary:**

Currently, there is a high prevalence of diseases that affect tooth‐supporting tissues (periodontal diseases) and negatively influence the oral health and quality of life of the adult population. These pathologies lead to movement of the teeth and impairment of chewing function, eventually resulting in the loss of teeth. In recent years, the concept of periodontal medicine has arisen and consists of studying how periodontal diseases can influence our general inflammatory system and how systemic inflammatory pathologies can affect our oral health. In the present study, we evaluate a group of women with polycystic ovary syndrome (PCOS), a condition characterized by alterations of sex hormones and lipid profile and weight gain (body mass index). Our results show a high prevalence of gum inflammation among women with PCOS, which affects the interaction of their leukocytes and endothelial cells. The leukocytes of these women are hyper‐responsive, presenting greater endothelial adhesion, lower flow velocity and enhanced rolling compared to those in a PCOS group without gum inflammation or controls. This study has generated a new line of research to analyze how neutrophils from patients with gingivitis exhibit hyperactivity, which promotes their interaction with the endothelium, thus contributing to the development of atherosclerotic disease.

## 1 INTRODUCTION

Recent research suggests an association between polycystic ovary syndrome (PCOS) and periodontal diseases such as gingivitis and periodontitis, thus highlighting the intricate interplay between oral health and systemic conditions.^1^ PCOS, a hormonal disorder prevalent among reproductive‐age women, is characterized by irregular menstrual cycles, excess androgen levels, and ovarian cysts.^2^ It is frequently associated with obesity, dyslipidemia, insulin resistance (IR), and hyperinsulinemia, all of which increase the risk of Type 2 diabetes and cardiovascular disease (CVD).^2^, ^3^, ^4^, ^5^ The pathogenesis of PCOS has been linked to an activation of the inflammatory response mediated by C‐reactive protein (CRP) and increased levels of inflammatory cytokines, blood lymphocytes, and monocytes, together with an enhanced expression of adhesion molecules and increased presence of markers of oxidative stress, such as lipid peroxidation and myeloperoxidase (MPO),^6^, ^7^ all of which promote the initial stages of atherosclerosis^8^—the main risk factor for CVD. Atherosclerosis, a chronic, progressive, inflammatory condition, is characterized by a prolonged asymptomatic phase known as subclinical atherosclerosis. This condition can progress to acute cardiovascular events, including myocardial infarction, unstable angina pectoris, and sudden cardiac death.^9^

Periodontal diseases are bacterial infections that affect the periodontium, an area of tissues including the gums, periodontal ligament, cement, and alveolar bone. There are two main types of periodontal diseases: gingivitis, characterized by inflammation of the gums, and periodontitis, which involves the irreversible destruction of the periodontal tissues and eventual loss of teeth.^10^ Similarly to the comorbidity of PCOS, several studies have highlighted an association between periodontitis and an increased risk of CVD,^11^, ^12^, ^13^ though evidence in the same respect regarding gingivitis is scarce. Previous studies have linked gingival inflammation to negative outcomes of endothelial dysfunction.^14^, ^15^ Moreover, a prospective 26‐year cohort study associated gingival inflammation with stroke,^16^ pointing further to a relationship between gingivitis and cardiovascular risk. There are several potential mechanisms linking periodontal diseases and CVD, including the release of cytokines, the production of toxins by oral bacteria, and the direct transfer of these compounds to the bloodstream.^17^ Recognition of lipopolysaccharide (LPS) by toll‐like receptor 4 (TLR4) and tumor necrosis factor alpha (TNFα) by the tumor necrosis factor receptor (TNFR) acts as a priming signal for innate phagocytes—mainly neutrophils—and induces nuclear factor kappa B (NF‐κB) and c‐Jun N‐terminal kinase (JNK) expression.^18^ JNK plays a pivotal role in activating the expression of proinflammatory genes, thereby promoting NF‐κB–mediated gene expression and amplifying the inflammatory response. Bacteria stimulate the release of TNFα, IL‐1, IL‐6, and IL‐8, which can invade the endothelial layer and promote the expression of chemokines and adhesion molecules.^19^ Additionally, the enhanced reactive oxygen species (ROS) production observed in the neutrophils of patients with periodontal diseases—particularly periodontitis^20^, ^21^—can contribute to increased cardiovascular risk due to the induction of endothelial dysfunction.^22^

Thus, the activation of several inflammatory pathways would appear to be an etiologic mechanism shared by PCOS and periodontal disease^1^, ^23^ and one that explains the increased cardiovascular risk associated with both pathologies. In light of this, we aimed to assess whether the presence of periodontal diseases—specifically gingivitis—contributes to an exacerbated inflammatory response and increased surrogate markers of atherosclerosis in a population of women with PCOS.

## 2 MATERIALS AND METHODS

This is a case–control study carried out at the University Hospital Doctor Peset, Valencia (Spain), between January 2019 and May 2022 in healthy subjects and women with PCOS between the ages of 18 and 45. The PCOS population was identified using the Rotterdam criteria;^24^ in other words, irregular ovulation (cycles over 35 days or under 26 days), elevated levels of free testosterone (>0.5 ng/dL), hirsutism, and polycystic ovaries. A group of healthy women was matched to the intervention group in terms of BMI and age.

Exclusion criteria for the study were other inflammatory conditions, recent use of antibiotics, taking anti‐inflammatory medications (NSAID or corticosteroids) during the last 6 months, cancerous or bone‐affecting pathologies, and conditions related to periodontal issues, such as diabetes or autoimmune diseases. It was also confirmed that none of the subjects had taken medication of any type during the previous semester. All the participants received detailed information highlighting the benefits and drawbacks of the study, and all signed an informed consent form and confidentiality commitment. This is a human observational study structured according to the STROBE (Strengthening the Reporting of Observational Studies in Epidemiology) guidelines and conducted in accordance with the Helsinki Declaration‐based ethical principles for medical research involving human subjects. All procedures were approved by the hospital's ethics committee (document of approval no. Ceim 31/19, registered under the number NCT06184412).

### 2.1 Clinical examination and medical history

Patients completed a health questionnaire regarding medication, pathologies, use of tobacco, frequency of tooth brushing, alcohol consumption, blood pressure, and anthropometric measurements. A complete periodontal examination was performed by a single experienced doctor (C.F.M‐A) following the consensus criteria.^25^, ^26^ Using a periodontal chart, all the subjects’ teeth were probed with a Williams‐type millimeter probe at six points: three buccal and three palatal. A radiographic exploration—orthopantomography—was carried out in all patients, and periapical radiographs were performed in those in whom periodontal pockets were detected. The following periodontal measures were recorded: percentage of bleeding on probing (BOP), millimeters of clinical attachment level (CAL), millimeters of probing pocket depth (PPD), percentage of loss of bone, number of teeth with PPD ≥ 4 mm, and number of teeth with CAL ≥ 4 mm. Plaque and calculus levels were evaluated using the Silness and Löe index^27^ and O'Leary index,^28^ respectively.

Gingivitis was defined by the presence of bleeding resulting from probing of the periodontal sulcus, performed with a Williams probe. To measure bleeding, we used the BOP percentage (defining gingivitis as BOP ≥ 10%), which was calculated with the following formula: total bleeding points/total probed points (6 × total number of teeth) × 100, in accordance with recent publications, seeking a consensus between the different scientific societies focused on periodontal pathologies.^25^

### 2.2 Blood sampling and isolation of neutrophils

A peripheral venous blood sample was taken after 12 h of fasting, during the follicular phase of the menstrual period. Biochemical parameters and sex hormonal profile were determined in the hospital's clinical analysis service according to standardized protocols. Neutrophils were isolated from EDTA‐anticoagulated peripheral blood by immunomagnetic separation*, following the manufacturer's protocol†, and then lysed for 15 min with radioimmunoprecipitation assay (RIPA) lysis buffer‡.

Aliquots of 25 μg of protein were resolved on 8%–16%‐gradient sodium dodecyl sulfate (SDS)‐polyacrylamide gels§ and transferred to nitrocellulose membranes. Target proteins were detected by incubating the membranes with rabbit polyclonal anti‐JNK‖ and mouse monoclonal anti‐actin¶ and the corresponding secondary antibodies. Enhanced chemiluminescence (ECL) Plus reagent# was used to detect the protein signal by chemiluminescence. The Fusion FX5 Acquisition System allowed visualization, and the one‐dimensional modeling code software** was used to quantify the band signal through densitometry.

### 2.3 Adhesion assay

A parallel plate flow chamber connected to an inverted microscope†† enabled the measurement of neutrophil–endothelial cell interactions in vitro. Before this, immortalized endothelial cells from human umbilical veins (HUVEC/TERT2)‡‡ were cultivated to complete confluence. Leukocytes were drawn across the HUVEC at a flow rate of 0.36 mL/min. A video camera§§ connected to the microscope permitted a view of the endothelial cells, and different leukocyte parameters (rolling velocity, rolling flux, and adhesion) were evaluated over 5 min.

### 2.4 Analysis of serum cytokines, cellular adhesion molecules, and glutathione

Serum levels of TNFα and MPO and the soluble intercellular adhesion molecule 1 (sICAM‐1), soluble vascular cell adhesion molecule 1 (sVCAM‐1), and soluble platelet selectin (sP‐selectin) were measured with a bead‐based immunoassay analyzer system‖‖ following the kit manufacturer's procedure¶¶. Total glutathione## content was evaluated following the kit manufacturer's procedure.

### 2.5 Statistical analysis

This study was designed to achieve a power of 80% and to detect significant differences of 20% with respect to the primary efficacy criterion, that is, leukocyte–endothelium interactions (measured in terms of adhesion of neutrophils), ≥5.5 cells/mm^2^, assuming a common standard deviation (SD) of four units based on a total of three groups. Under these premises, at least 12 subjects per group were considered. The normal distribution of the samples was confirmed using the Kolmogorov–Smirnov test or the Saphiro–Wilk test. Normally and non‐normally distributed data were expressed as mean ± SD and median (25^th^–75th percentiles), respectively. Qualitative data were expressed as percentages. Data were analyzed using an unpaired Student's *t* test or one‐way analysis of variance (ANOVA) for parametric data and a Mann–Whitney *U* test or Kruskal–Wallis test for nonparametric data. The strength of the association between variables was measured with Pearson's or Spearman's correlation coefficient. To predict the value of a variable based on another variable, linear regression analysis was employed. Differences were considered significant at a *p* value <0.05, with a confidence interval of 95%. Analyses were performed with the Statistical Package for the Social Sciences***.

## 3 RESULTS

Our study population included a total of 104 women with an average age of 28 years and a BMI of 25.3 kg/m^2^. The population was divided into three groups: a control group with no pathology (*n* = 36) and two groups of women with PCOS, one with (PCOS+, *n* = 24) and one without gingivitis (PCOS−, *n* = 44). As shown in Table 1, anthropometric variables (age, BMI, systolic blood pressure [SBP], or diastolic blood pressure [DBP]) did not differ significantly among the groups. Waist circumference was higher in PCOS patients (71.5 [67.1, 77.8] vs. 75.5 [70.0, 83.0], *p* < 0.05), specifically among those with gingivitis. As expected, the hydrocarbonated metabolism was altered in the PCOS groups, in which levels of insulin and homeostasis model assessment of insulin resistance (HOMA‐IR) were increased (*p* < 0.05), as well as other surrogate markers of IR, such as complement factor c3 (C3c) and retinol‐binding protein 4 (RBP4) (*p* < 0.001). Total cholesterol (179 ± 27 vs. 192 ± 36, *p* < 0.05), low‐density lipoprotein cholesterol (LDL‐C) (101 ± 23 vs. 113 ± 32, *p* < 0.05), and triglycerides (61 [46, 70] vs. 79 [54, 120]), *p* < 0.01) were higher in PCOS patients and still higher in those with gingivitis, while high‐density lipoprotein cholesterol (HDL‐C) was similar in all three groups.

**TABLE 1:** Anthropometric, biochemical, and administered medication in study population according to presence or absence of gingivitis.

Variable	Control	PCOS−	PCOS+
*n*	36	44	24
Age (years)	28.9 ± 6.3	27.4 ± 5.7	28.7 ± 5.9
BMI (kg/m^2^)	24.9 ± 3.8	25.3 ± 5.2	26.0 ± 4.7
Waist (cm)	71.5 (67.1, 77.8)	74.5 (70.0, 80.8)	78.0 (73.5, 86.3)^*^
SBP (mm Hg)	111 ± 14	109 ± 11	115 ± 11
DBP (mm Hg)	76 ± 8	78 ± 10	81 ± 9
Glucose (mg/dL)	88 ± 8	87 ± 10	89 ± 8
Insulin (μUI/mL)	6.71 ± 2.39	9.65 ± 7.78^*^	9.74 ± 4.71^*^
HOMA‐IR	1.46 ± 0.57	1.98 ± 1.57^*^	2.15 ± 1.13^*^
Cholesterol (mg/dL)	179 ± 27	192 ± 39	193 ± 30
HDL‐C (mg/dL)	65 ± 13	63 ± 17	57 ± 13
LDL‐C (mg/dL)	101 ± 23	111 ± 35	118 ± 26^*^
Triglycerides (mg/dL)	61 (46, 70)	69 (51, 120)	95 (63, 122)^*^
hsCRP (mg/L)	0.81 (0.34, 3.10)	3.34 (0.62, 6.68)	3.16 (1.12, 4.19)
C3c (mg/dL)	105 ± 17	121 ± 23^*^	122 ± 17^*^
RBP4 (mg/dL)	1.76 ± 0.42	2.22 ± 0.60^*^	2.13 ± 0.44^*^
FSH (mUI/dL)	4.41 ± 2.07	3.61 ± 2.09	4.08 ± 1.86
LH (mUI/dL)	5.08 ± 4.52	4.48 ± 4.64	3.84 ± 2.11
Estradiol (pg/mL)	86 ± 73	49 ± 54^*^	56 ± 52
Testosterone (ng/dL)	0.381 ± 0.238	0.436 ± 0.175	0.379 ± 0.125
Androstenedione (ng/mL)	2.99 ± 1.33	3.21 ± 1.28	3.04 ± 1.29
DEAHS (μg/dL)	231 ± 101	285 ± 115^*^	249 ± 106^**^
17OH‐P (ng/dL)	1.96 ± 1.92	1.28 ± 1.72	1.12 ± 0.91
SHBG (μg/dL)	85 ± 70	162 ± 162^*^	123 ± 107
FAI (%)	1.98 ± 1.18	2.54 ± 3.83	2.43 ± 2.53
Prolactin (ng/dL)	22.8 ± 11.8	18.4 ± 10.8	22.5 ± 12.1
Medication *n* (%)			
Oral contraceptives	3 (2.9)	21 (20.2)^*^	12 (11.5)
Metformin	0 (0)	10 (9.6)^*^	3 (2.9)^*^

*Note*: Data are presented as mean ± SD. Waist, triglycerides, and hsCRP are represented as median and interquartile range (25% and 75% percentile). Data were compared by one‐way analysis of variance or Kruskal–Wallis followed by Student–Newman–Keuls post hoc test.

Abbreviations: 17OH‐P, 17‐hydroxyprogesteron; BMI, body mass index; C3c, complement factor c3; DBP, diastolic blood pressure; DEAHS, dehydroepiandrosterone‐sulfate; FAI, free androgen index; FSH, follicle‐stimulating hormone; HDL‐C, high‐density lipoprotein cholesterol; HOMA‐IR, homeostasis model assessment of insulin resistance (fasting insulin (μU/mL) × fasting glucose (mg/dL))/405); hsCRP, high‐sensitivity C‐reactive protein; LDL‐C, low‐density lipoprotein cholesterol; LH, luteinizing hormone; PCOS−, polycystic ovary syndrome without gingivitis; PCOS+, polycystic ovary syndrome with gingivitis; RBP4, retinol‐binding protein 4; SBP, systolic blood pressure; SHBG, sex hormone‐binding globulin.

^*^ *p* < 0.05 when compared with those of control group.

^**^ *p* < 0.05 when compared with PCOS− group.

Sexual hormone profiles revealed a lower concentration of estradiol and 17‐hydroxyprogesteron (17OH‐P) (86 ± 73 vs. 52 ± 53 and 1.96 ± 1.92 vs. 1.22 ± 1.46, respectively; *p* < 0.05 for both) and higher levels of sex hormone‐binding globulin (SHBG) (85 ± 70 vs. 148 ± 145, *p* < 0.01) in women with PCOS, which was probably associated with the higher prevalence of women taking oral contraceptives in this group. Metformin was also more frequently prescribed among our subjects with PCOS due to its effect as a sensitizer of insulin action (Table 1).

Moreover, the evaluation of periodontal parameters revealed a marked increase in BOP, plaque, and calculus index in patients with gingivitis, while there were no significant differences in the remaining variables (Table 2).

**TABLE 2:** Periodontal parameters and treatment of study population according to presence or absence of gingivitis.

Variable	Control	PCOS−	PCOS+
PPD (mm)	1.651 ± 0.421	1.564 ± 0.330	1.660 ± 0.329
CAL (mm)	1.657 ± 0.424	1.567 ± 0.333	1.607 ± 0.475
BOP (%)	1.935 ± 2.336	3.211 ± 2.644	13.636 ± 3.375^[Link]^, ^[Link]^
Plaque index	0.423 ± 0.408	0.598 ± 0.472	0.843 ± 0.608^[Link]^, ^[Link]^
Calculus index	0.021 ± 0.053	0.022 ± 0.066	0.165 ± 0.436^[Link]^, ^[Link]^
Teeth loss (*n*)	0.472 ± 1.764	0.682 ± 1.394	0.458 ± 1.062
Teeth with PPD ≥ 4 mm, *n*	0.167 ± 0.561	0.295 ± 1.00	0.542 ± 0.932
Teeth with CAL ≥ 4 mm, *n*	0.028 ± 0.167	0.318 ± 1.051	0.333 ± 1.434
Periodontal treatment, *n* (%)			
Never	7 (6.7)	6 (5.8)	3 (2.9)
Tartrectomy	29 (27.9)	35 (33.7)	19 (18.3)
SRP	0 (0)	3 (2.9)	2 (1.9)

*Note*: Data are expressed as mean ± SD. Data were compared by one‐way analysis of variance or Kruskal–Wallis followed by Student–Newman–Keuls post hoc test.

Abbreviations: BOP, bleeding on probing; CAL, clinical attachment level; PCOS−, polycystic ovary syndrome without gingivitis; PCOS+, polycystic ovary syndrome with gingivitis; PPD, probing pocket depth; SRP, periodontal scaling and root planing.

^*^ *p* < 0.05 when compared with those of control group.

^**^ *p* < 0.05 when compared with PCOS− group.

**FIGURE 1:**
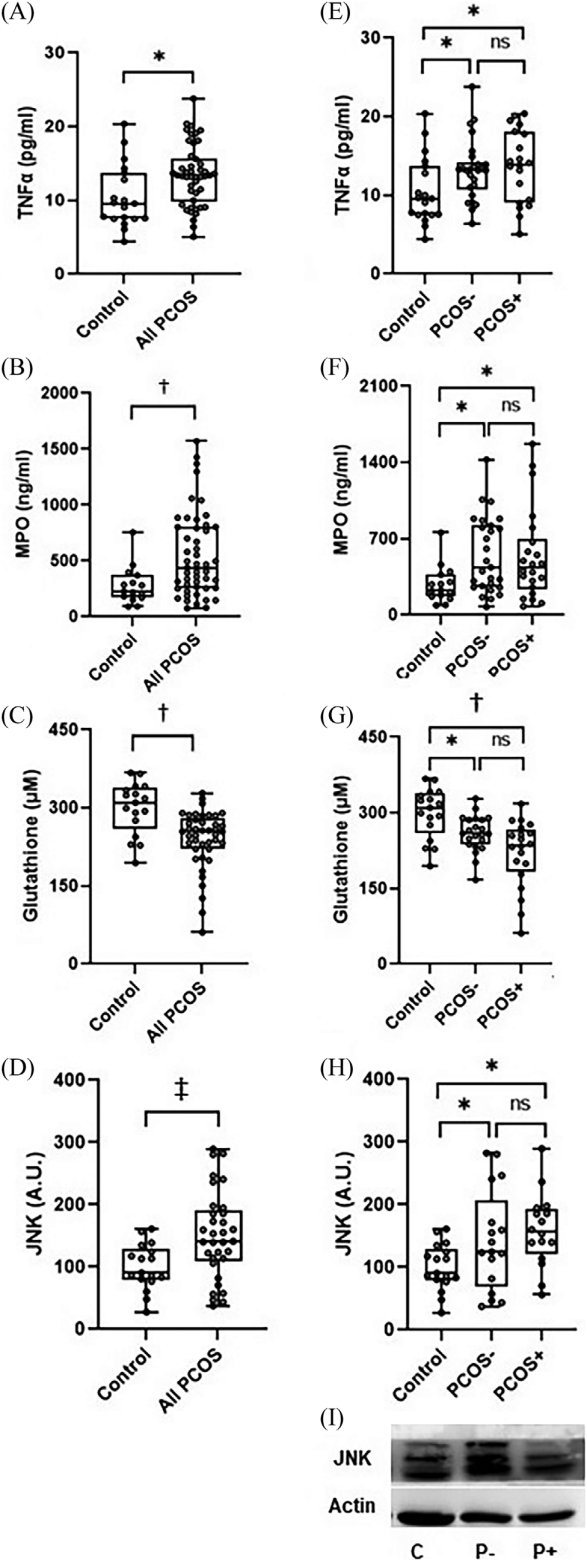
Inflammatory and oxidative stress markers in study population according to presence or absence of gingivitis. Systemic concentration of TNFα considering controls and PCOS patients as a whole (*n* = 19 and *n* = 46, respectively) (A) or control, PCOS−, or PCOS+ (*n* = 19, *n* = 26, and *n* = 20, respectively) (E). Systemic concentration of MPO considering control and PCOS patients as a whole (*n* = 16 and *n* = 48, respectively) (B) or control, PCOS−, or PCOS+ (*n* = 16, *n* = 27, and *n* = 21, respectively) (F). Systemic concentration of glutathione considering control and PCOS patients as a whole (*n* = 17 and *n* = 43, respectively) (C) or control, PCOS−, or PCOS+ (*n* = 17, *n* = 21, and *n* = 22, respectively) (G). Levels of JNK protein expression in leukocytes considering control and PCOS patients as a whole (*n* = 17 and *n* = 32, respectively) (D) or control, PCOS−, or PCOS+ (*n* = 17, *n* = 17, and *n* = 15, respectively) (H) and representative Western blot images (I). Data are presented as box and whisker plots. Data of patients with PCOS and controls were compared with unpaired Student *t* test; to analyze differences between three groups, one‐way analysis of variance was applied followed by Student–Newman–Keuls post hoc test. JNK, c‐Jun N‐terminal kinase; MPO, myeloperoxidase; ns, not significant; PCOS, polycystic ovary syndrome; PCOS−, PCOS without gingivitis; PCOS+, PCOS with gingivitis; TNFα, tumor necrosis factor alpha. *p < 0.05; ^‡^ *p* < 0.01; ^†^ *p* < 0.001.

**FIGURE 2:**
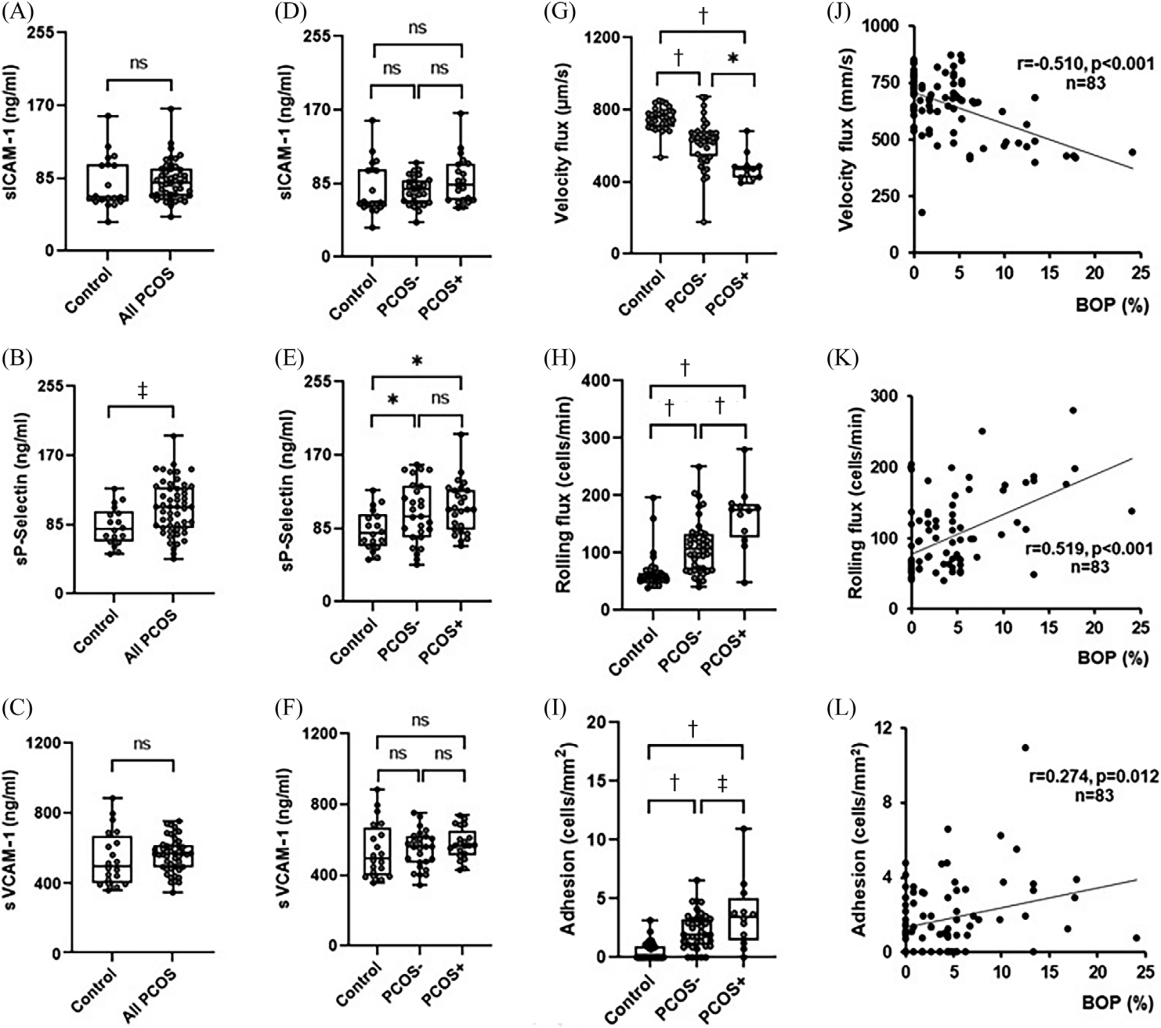
Evaluation of leukocyte–endothelial cell interactions and serum levels of CAMs in study population according to presence or absence of gingivitis. Systemic concentration of sICAM‐1 considering controls and PCOS patients as a whole (*n* = 19 and *n* = 48, respectively) (A) or control, PCOS−, or PCOS+ (*n* = 19, *n* = 27, and *n* = 21, respectively) (D). Systemic concentration of sP‐selectin considering control and PCOS patients as a whole (*n* = 16 and *n* = 48, respectively) (B) or control, PCOS−, or PCOS+ (*n* = 16, *n* = 27, and *n* = 21, respectively) (E). Systemic concentration of sVCAM‐1 considering control and PCOS patients as a whole (*n* = 17 and *n* = 48, respectively) (C) or control, PCOS−, or PCOS+ (*n* = 17, *n* = 27, and *n* = 21, respectively) (F). Leukocyte velocity flux (G), rolling flux (H), and leukocyte adhesion (I) considering controls, PCOS−, or PCOS+ (*n* = 31, *n* = 40, and *n* = 12, respectively). Correlation analysis of BOP and velocity flux (J), rolling flux (K), and leukocyte adhesion (L). Data are presented as box and whisker plots. Data of patients with PCOS and controls were compared with unpaired Student *t* test; to analyze differences between three groups, one‐way analysis of variance was applied followed by Student–Newman–Keuls post hoc test. BOP, bleeding on probing; CAM, cellular adhesion molecule; ns, not significant; PCOS, polycystic ovary syndrome; PCOS−, PCOS without gingivitis; PCOS+, PCOS with gingivitis; sICAM‐1, soluble intercellular adhesion molecule 1; sP‐selectin, soluble platelet selectin; sVCAM‐1, soluble vascular cell adhesion molecule 1. ^*^ *p* < 0.05; ^**^ *p* < 0.01; ^***^ *p* < 0.001.

As expected, PCOS patients displayed higher levels of proinflammatory markers, such as high‐sensitivity C‐reactive protein (hsCRP) (0.81 [0.34, 3.10] vs. 316 [0.66, 5.32], *p* < 0.05) (Table 1) and TNFα (*p* < 0.05) (Figure 1A). Additionally, systemic markers of prooxidants, such as MPO (Figure 1B), were increased and antioxidant systems (glutathione) were reduced among these patients (Figure 1C) (*p* < 0.001 for both), suggesting an oxidative stress status. However, gingivitis did not seem to contribute significantly to these circumstances (Figure 1E–1G). When we explored the inflammatory mediators of neutrophils, we observed a significantly higher expression of JNK in women with PCOS (Figure 1D), independently of the presence or absence of gingivitis (Figure 1H,I). Under these conditions, activated neutrophils are more prone to interact with the endothelium, and accordingly, we observed impaired adhesion under flow conditions, manifested by a progressive reduction in rolling velocity in the PCOS groups, which was more evident among women with gingivitis (Figure 2G). A similar response was observed with rolling flux (Figure 2H) and cellular adhesion (Figure 2I). These alterations were accompanied by a significant increase in sP‐selectin in the case of the PCOS groups as a whole (Figure 2B,E), but we did not detect any changes in sICAM‐1 (Figure 2A,D) or sVCAM‐1 (Figure. 2C,F). As neutrophil–endothelial cell interactions were statistically different among the three groups, we analyzed associations with the other parameters studied. Strikingly, we discovered that rolling velocity (*r* = −0.510, *p* < 0.001) (Figure 2J), rolling flux (*r* = 0.519, *p* < 0.001) (Figure 2K), and adhesion (*r* = 0.274, *p* = 0.012) (Figure 2L) correlated with BOP. The correlation analysis also demonstrated that rolling velocity correlated negatively with triglycerides, C3c, RBP4, calculus index, TNFα, and sP‐selectin and positively with estradiol (Table 3). In the multivariable regression model, the association of rolling velocity with correlated variables was evaluated as a potentially independent predictor using the stepwise method. The results revealed that BOP (*β* = −0.662) and TNFα (*β* = −0.280) were independently associated with rolling velocity, thus explaining 55.9% of the dependent variable (Table 4). In addition to BOP, rolling flux correlated positively with triglycerides, number of teeth lost, TNFα, sVCAM‐1, and sP‐selectin and negatively with estradiol and glutathione (Table 3). Multivariable analysis showed that BOP (*β* = −0.560) and number of teeth lost (*β* = −0.301) were independently associated with rolling flux, explaining 44.7% of the dependent variable. And finally, cellular adhesion correlated positively with BOP, cholesterol, LDL‐C, C3c, RBP4, calculus index, and number of teeth with CAL ≥ 4 mm (Table 2). Of these variables, number of teeth with CAL ≥ 4 mm (*β* = 0.277), BOP (*β* = 0.222), and cholesterol (*β* = 0.236) contributed independently to cellular adhesion, explaining 25.8% of the dependent variable.

**TABLE 3:** Correlation coefficients of leukocyte–endothelium cell interactions and biochemical and periodontal parameters, endothelial dysfunction, and inflammatory variables.

Variable	Velocity flux (mm/s)	Rolling flux (cells/min)	Adhesion (cells/mm^2^)
Cholesterol (mg/dL)	–	–	0.333^*^ (84)
LDL‐C (mg/dL)	–	–	0.309^*^ (84)
Triglycerides (mg/dL)	−0.246^*^ (83)	0.257^*^ (83)	–
C3c (mg/dL)	−0.389^*^ (82)	–	0.299^*^ (83)
RBP4 (mg/dL)	−0.277^**^ (81)	–	0.359^*^ (82)
Estradiol (pg/mL)	0.244^**^ (83)	−0.288^**^ (83)	–
Calculus index	−0.272^**^ (83)	–	0.299^*^ (84)
Teeth loss (*n*)	–	0.239^**^ (83)	–
Teeth with CAL ≥ 4 mm, *n*	–	–	0.298^*^ (84)
TNFα (pg/mL)	−0.460^*^ (52)	0.334^**^ (52)	–
Glutathione (μM)	0.355^**^ (47)	−0.338^**^ (47)	–
sVCAM‐1 (ng/mL)	–	0.271^**^ (54)	–
sP‐Selectin (ng/mL)	−0.300^**^ (53)	0.289^**^ (53)	–

*Note*: Only variables that show significant differences (*p* < 0.05, *p* < 0.01) are represented in the table. Data are expressed as Pearson's or Spearman's coefficient correlation for parametric and nonparametric data, respectively. Sample size is shown in parentheses.

Abbreviations: C3c, complement factor c3; CAL, clinical attachment level; LDL‐C, low‐density lipoprotein cholesterol; RBP4, retinol‐binding protein 4; sP‐selectin, soluble platelet selectin; sVCAM‐1, vascular cell adhesion molecule 1; TNFα, tumor necrosis factor alpha.

^*^ *p* < 0.01.

^**^ *p* < 0.05.

**TABLE 4:** Simple linear regression model of parameters of leukocyte–endothelium cell interaction as dependent variables.

Dependent variable	Independent variable	*β*	Standard error	Beta coefficient	Adjust *R* ^2^	*p* value
Velocity flux					0.559	<0.001
	Constant	794.9	36.6			<0.001
	BOP	−12.9	2.1	−0.662		<0.001
	TNFα	−8.11	2.90	−0.280		0.008
Rolling flux					0.417	<0.001
	Constant	79.6	7.7			<0.001
	BOP	5.10	0.99	0.560		<0.001
	Teeth loss	14.8	5.4	0.301		0.008
Adhesion					0.258	<0.001
	Constant	−2.74	1.09			0.014
	Teeth with CAL ≥ 4 mm, *n*	0.631	0.223	0.277		0.006
	BOP	0.085	0.037	0.222		0.026
	Cholesterol	0.014	0.006	0.236		0.037

Abbreviations: BOP, bleeding on probing; CAL, clinical attachment level, TNFα, tumor necrosis factor alpha.

## 4 DISCUSSION

In the present study, we demonstrate an increase of subclinical markers of atherosclerosis mediated by an alteration of neutrophil–endothelium cell interactions in subjects with PCOS, in whom neutrophil rolling velocity decreased and rolling flux and cellular adhesion increased with the presence of gingivitis. Moreover, this response was dependent on BOP and was associated with soluble adhesion molecules, impaired redox status, and a marked proinflammatory profile.

Women with PCOS often exhibit increased oxidative stress and chronic low‐grade inflammation. In this context, total and mitochondrial ROS, MPO, and hsCRP and inflammatory cytokines such as IL6 and TNFα.^29^, ^30^ are reported to be increased in PCOS patients, which our present findings confirm. Recent studies indicate that women with PCOS are more susceptible to periodontal issues. Chronic low‐grade inflammation, a common feature in both PCOS and periodontal diseases, maybe a bridge connecting these seemingly disparate conditions.^1^, ^31^, ^32^

Innate immunity is heavily influenced by neutrophils—the most abundant type of leukocyte in the blood and gingival tissues—in response to plaque accumulation.^33^ Under homeostatic conditions, neutrophils typically respond to microbial communities in a way that resolves inflammation. However, an imbalance in the microbial ecosystem heightens neutrophil activity, which perpetuates the inflammatory response. In such conditions, neutrophils employ potent antimicrobial mechanisms that can damage nearly all cellular components, such as phagocytosis, neutrophil extracellular trap (NET), vesicular antimicrobial peptide release, and ROS generation via MPO.^34^, ^35^ This dual action of neutrophils is pivotal in the immune response and contributes to the progression of various pathologies, including periodontal diseases^36^ and CVD.^37^ This latter role is exerted through several mechanisms: generation of dysfunctional lipoproteins, reduced nitric oxide (NO) availability, endothelial dysfunction, and atherosclerotic plaque instability. In the present study, we have observed elevated levels of MPO and a decrease in glutathione content among our PCOS population, suggesting a state of oxidative stress accompanied by altered subclinical atherosclerotic markers (e.g., increased sP‐selectin levels and neutrophil–endothelial cell interactions), findings that are in line with previous reports.^25^, ^34^ Interestingly, we did not find gingivitis to be associated with these alterations, since MPO did not vary between the two groups of women with PCOS.

Neutrophil activation has also been associated with an increase in JNK signaling. In an in vitro model, Khan et al.^18^ observed activation of the LPS–TLR4–JNK axis in human neutrophils, describing it as a molecular switch that senses LPS concentration and bacterial loads that can induce NETosis. However, we did not detect significant differences between PCOS groups, suggesting that this axis was not activated by gingivitis, perhaps due to the low LPS load present in the systemic circulation. Indeed, of the total number of samples included in the assay, LPS was detectable in only 10% (data not shown). These results imply that neither the activation of the JNK pathway nor the release of MPO plays a main role in the activation of neutrophils in gingivitis (manifested by reduced rolling velocity and augmented rolling flux and cellular adhesion). It would seem there are alternative pathways of neutrophil activation involved in the higher risk of subclinical atherosclerosis observed in patients with gingivitis.

Obviously, our patients with gingivitis exhibited altered periodontal parameters, primarily plaque index and BOP, the latter of which was found to be independently associated with neutrophil–endothelium cell interactions (velocity, rolling, and adhesion). BOP is a clinical marker of the gingival inflammatory response triggered by bacterial pathogens and orchestrated by neutrophils. In addition to classic priming signaling of neutrophils such as LPS, cytokines, and chemokines, it has been shown that neutrophil priming occurs under conditions of metabolic dysregulation. Mechanistically, hypercholesterolemia and hyperglycemia can promote chronic neutrophil‐mediated inflammation.^38^, ^39^, ^40^ In hypercholesterolemia, there is elevated production of neutrophils, which display increased mobilization from the bone marrow, circulating in a prime state characterized by enhanced responsiveness of ROS and MPO to secondary stimuli.^39^, ^41^ Within this context, Obama et al. reported that oxidized low‐density lipoprotein (oxLDL) acts synergistically with neutrophils to form NET and promote vascular endothelial inflammation.^42^ We found higher levels of LDL and triglycerides specifically in PCOS patients with gingivitis, which points to a role for cholesterol as an independent variable in cellular adhesion to the endothelium and is perhaps suggestive of a prime phenotype. Indeed, it has recently been demonstrated that experimental gingivitis in humans induces hyper‐reactivity of peripheral neutrophils under stimulated conditions, leading to an increase in ROS, NET, and chemotaxis.^43^ We believe our study goes one step further since it evaluates the impact of gingivitis on circulating neutrophils and their interaction with the endothelium as a surrogate marker of atherosclerosis. In fact, until now, only two studies have addressed the relationship between gingival inflammation and endothelial function in humans. Carallo et al. reported a negative effect of gingival inflammation on flow‐mediated dilatation in a healthy population without periodontal disease.^14^, while Eberhard et al. observed increased expression of inflammatory mediators, enhanced adherence of monocytes to endothelial cells, and increased foam cell formation after oxLDL uptake.^15^ Our present findings are in line with these two previous reports since we describe reduced velocity flux and increased rolling flux and cellular adhesion in a population of women with PCOS and gingivitis, reaffirming that gingival inflammation—determined by BOP—is partially responsible for the higher risk of atherosclerosis among these subjects. Future research needs to explore the intracellular signaling involved in this neutrophil hyper‐reactivity.

Our study has some limitations, including the relatively small sample of women with gingivitis, though it was supported by sample size calculation. In addition, we did not determine the presence (or absence) of the atherosclerotic plaque or blood count and did not assess cytokines in the gingival crevicular fluid. Finally, the cross‐sectional nature of this study limits its interpretability.

## 5 CONCLUSION

To conclude, our findings highlight that periodontal tissue inflammation has systemic effects that predispose patients to an exacerbated innate immune response, though they do not rule out the involvement of hypercholesterolemia. Moreover, this hyper‐activation of neutrophils promotes their interaction with the endothelium, thereby increasing the risk of atherosclerosis. This indicates that peripheral neutrophils can respond synergistically to simultaneous triggers of inflammation and therefore may explain the connection between periodontal disease and other inflammatory conditions such as PCOS. This implies broader implications of periodontal disease beyond oral health and points the way to novel strategies for addressing systemic inflammatory conditions linked to periodontal diseases.

### 5.1 Clinical relevance

Although gingivitis is a reversible condition that can be effectively managed and resolved, it is imperative to acknowledge that it has potentially far‐reaching consequences for systemic health, particularly among individuals with pre‐existing medical conditions such as PCOS. Therefore, it is essential to raise awareness among the population about the importance of good oral hygiene practices for preventing future systemic diseases and, more specifically, the development of atherosclerotic cardiovascular disease.

## AUTHOR CONTRIBUTIONS

All authors have made substantial contributions to the conception and design of the study. Cecilia Fabiana Márquez‐Arrico, Francisco Javier Silvestre, Meylin Fernández‐Reyes, Sandra López‐Domènech, José María Montiel‐Company, Víctor M. Víctor, and Milagros Rocha have been involved in data collection and data analysis. Cecilia Fabiana Márquez‐Arrico, Francisco Javier Silvestre, Meylin Fernández‐Reyes, Sandra López‐Domènech, Jonathan Hermenejildo, Zaida Abad‐Jiménez, Javier Silvestre‐Rangil, Pablo Fernández‐Collazo, Carlos Morillas, José María Montiel‐Company, Víctor M. Víctor, and Milagros Rocha have been involved in data interpretation and drafting the manuscript and revising it critically and have given final approval of the version to be published.

## CONFLICT OF INTEREST STATEMENT

The authors declare that they do not have any conflicts of interest to disclose.

## Data Availability

The data that support the findings of this study are available on request from the corresponding author. The data are not publicly available due to privacy or ethical restrictions.

## Footnotes

* MACSprep Leukocyte Isolation Kit; Milteny Biotech, Germany.

† Thermo Fisher Scientific, Waltham, Massachusetts, USA.

‡ Invitrogen, Carlsbad, California, USA.

§ Calbiochem, San Diego, California, USA.

‖ Cell Signaling Technology, Danvers, Massachusetts, USA.

¶ Healthcare, Uppsala, Sweden.

# Vilbert Lourmat, Marne‐la‐Vallée, France.

** Bio1D software version 15.03a; Nikon Eclipse TE 2000‐S.

†† ATCC, Manassas, Virginia, USA.

‡‡ Sony Exware HAD, Cologne, Germany.

§§ Sony Exware HAD, Cologne, Germany.

‖‖ Luminex Thermo Fisher Scientific, Waltham, Massachusetts, USA.

¶¶ Milliplex MAP; Millipore Corporation, Billerica, Massachusetts, USA.

## Cayman Chemical, Ann Arbor, Michigan, USA.

*** SPSS 22.0; Statistics Inc., Chicago, Illinois, USA.
